# Repellent active ingredients encapsulated in polymeric nanoparticles: potential alternative formulations to control arboviruses

**DOI:** 10.1186/s12951-022-01729-7

**Published:** 2022-12-10

**Authors:** Daniele Carvalho Abrantes, Carolina Barbara Rogerio, Estefânia Vangelie Ramos Campos, Tais Germano-Costa, Aryane Alves Vigato, Ian Pompermeyer Machado, Anderson Ferreira Sepulveda, Renata Lima, Daniele Ribeiro de Araujo, Leonardo Fernandes Fraceto

**Affiliations:** 1 São Paulo State University (UNESP), Institute of Science and Technology, Avenida Três de Março 511, Alto da Boa Vista, Sorocaba, São Paulo 18087-180 Brazil; 2 Laboratory of Bioactivity Assessment and Toxicology of Nanomaterials, University of Sorocaba, Sorocaba, São Paulo Brazil; 3 Human and Natural Sciences Center, Federal University of ABC, Santo André, São Paulo 09210-580 Brazil

**Keywords:** Arboviruses, Insect repellent, PCL nanoparticle, Geraniol, IR3535

## Abstract

Dengue, yellow fever, Chinkungunya, Zika virus, and West Nile fever have infected millions and killed a considerable number of humans since their emergence. These arboviruses are transmitted by mosquito bites and topical chemical repellents are the most commonly used method to protect against vector arthropod species. This study aimed to develop a new generation of repellent formulations to promote improved arboviruses transmission control. A repellent system based on polycaprolactone (PCL)-polymeric nanoparticles was developed for the dual encapsulation of IR3535 and geraniol and further incorporation into a thermosensitive hydrogel. The physicochemical and morphological parameters of the prepared formulations were evaluated by dynamic light scattering (DLS), nano tracking analysis (NTA), atomic force microscopy (AFM). In vitro release mechanisms and permeation performance were evaluated before and after nanoparticles incorporation into the hydrogels. FTIR analysis was performed to evaluate the effect of formulation epidermal contact. Potential cytotoxicity was evaluated using the MTT reduction test and disc diffusion methods. The nanoparticle formulations were stable over 120 days with encapsulation efficiency (EE) of 60% and 99% for IR3535 and geraniol, respectively. AFM analysis revealed a spherical nanoparticle morphology. After 24 h, 7 ± 0.1% and 83 ± 2% of the GRL and IR3535, respectively, were released while the same formulation incorporated in poloxamer 407 hydrogel released 11 ± 0.9% and 29 ± 3% of the loaded GRL and IR3535, respectively. GRL permeation from PCL nanoparticles and PCL nanoparticles in the hydrogel showed similar profiles, while IR3535 permeation was modulated by formulation compositions. Differences in IR3535 permeated amounts were higher for PCL nanoparticles in the hydrogels (36.9 ± 1.1 mg/cm^2^) compared to the IR3535-PCL nanoparticles (29.2 ± 1.5 mg/cm^2^). However, both active permeation concentrations were low at 24 h, indicating that the formulations (PCL nanoparticles and PCL in hydrogel) controlled the bioactive percutaneous absorption. Minor changes in the stratum corneum (SC) caused by interaction with the formulations may not represent a consumer safety risk. The cytotoxicity results presented herein indicate the carrier systems based on poly-epsilon caprolactone (PCL) exhibited a reduced toxic effect when compared to emulsions, opening perspectives for these systems to be used as a tool to prolong protection times with lower active repellent concentrations.

**Supplementary Information:**

The online version contains supplementary material available at 10.1186/s12951-022-01729-7.

## Introduction

Arboviruses, or arthropod-borne viruses, are diseases transmitted by blood-feeding arthropod saliva and replicated/amplified in vertebrates hosts. Approximately 530 types of these viruses have been identified, of which 100 can affect humans and originate from families such as Togaviridae, Flaviviridae, Bunyaviridae, Reoviridae, Rhabdoviridae, Orthomyxoviridae, and Asfarviridae [1, 2].

In humans these viruses are typically transmitted by mosquito bites, with dengue, yellow fever, chinkungunya, zika virus and West Nile fever as representative examples. These diseases have infected millions and kill a considerable number of humans since their emergence. Arboviruses are present worldwide as consequence of rapid urbanization, climate change, and intense population flow associated with travel and migration [1, 3, 4].

Only a few vaccines have been developed for these diseases, indicating that other prevention methods are important. Protection measures including the use clothes, avoiding activities outdoor during times with higher mosquitoes activity, protective screens, and chemical repellents have been used for arboviruses prevention [5].

In addition, the use of repellents as protection for humans can be obtained from natural or synthetic sources. Ideally, these repellants should be safe for humans, with low toxicity, chemically stability, low odor, and bug bite prevention properties that transform skin into an undesirable place for the insect vectors [6, 7].

The most common synthetic repellents include N,N-diethyl-3-methylbenzenamide (DEET), 1-piperidinecarboxylic acid 2-(2-hydroxyethyl)-1-methylpropylester (picaridin), and ethyl 3-]acetyl(butyl)amino]propanoate (IR3535). DEET is the gold standard synthesized in 1956 in the USA by the Department of Agriculture and has been applied worldwide used against flies, mosquitoes and fleas in formulations as aerosols, creams and sprays. Although the US Environmental Protection Agency determined in 2014 that DEET poses no risks to humans, the environment, and non-target species [6, 8, 9], it can cause itchy skin, inflammation, and seizures in children [10, 11].

Picaridin was developed in the 1990s by Bayer AG and is commercialized in 50 countries as a promising compound against flies, mosquitoes, bees, fleas, and ticks in sprays, lotions, and wet wipe formulations. It is indicated for use in children > 2 years old in concentrations ranging from 7 to 25 vol% presenting light stability, resistance against water and sweat, and skin compatibility [9, 12, 13]. IR3535 was developed by Merck and commercialized in USA in the 1970s. It is commercially available as aerosols, sprays, and lotions in concentrations up to 30% for use against flies, mosquitoes, ticks, lice, wasps, and bees. IR3535 is recommended for use in children < 2 years old and pregnant women in concentrations up to 12.5% [7, 14, 15].

DEET exhibits demonstrated cytotoxicity by decreasing in cell viability in human BE(2)-M17 cell line at 500–4000 mg/L with increased apoptotic protein expression that was not dose dependent [16]. Another study showed that picaridin and DEET pose no toxicological risks for humans, characterized by margin of exposure (MOE) and no-observed-effect-levels (NOEL) results [17]. DEET was also studied in red blood cells and induced hemolysis when present at 4–5 mM, causing premature eryptosis death, cell shrinkage, membrane damage, and altered Ca^2+^ influx [10].

Natural repellent alternatives based on essential oils are typically extracted from plants and exhibit low toxicity consisting mainly of volatile organic compounds. In particular, lemon eucalyptus and citronella oils are commonly used with major active components including p-Menthane-3,8-diol (PMD) and trans-3,7-dimethyl-2,6-octadiene-1-ol (geraniol), respectively [7, 18]. PMD can be extracted from leaves of the *Corymbia citriodora* tree and is the only natural repellent endorsed by the Centers of Disease Control and Prevention, with comparable efficacy as DEET. Geraniol is a monoterpene found in several essential oils but primarily isolated from Palmarosa oil that can also be used as a repellent [19–22]

The mixture of different repellents can produce a formulation with improved repellent properties by combining various properties. Alavez-rosas et al. [23] studied citronella, mint, rosemary, and clove oils against *Aedes aegypti* and observed that a ternary mixture of citronella:mint:clove (1:1:1) showed similar repellency as 5 and 10% commercial DEET. Noosidum et al. [24] studied oils extracted from *Litsea cubeba*, *Litsea salicifolia*, and *Melaleuca leucadendron* against *Ae. aegypti*. The mixture of *Litsea cubeba* and *Litsea salicifolia* (0.075%) showed pronounced synergistic effects against the mosquitoes and non-contact repellency, which were like the DEET response.

Although natural repellents exhibit many benefits, essential oils are easily volatilized, and their instability necessitates the development of novel technologies that can control their release when applied topically. Nanotechnology is a promising strategy for pollution reduction, sustainability, as well as biocompatible and biodegradable material production for formulations containing essential oils. These formulations can protect the actives and decrease environmental losses through encapsulation of the repellents, providing prolonged active ingredient release [25, 26]

Polymeric nanoparticles are widely used as topical drug delivery systems, especially nanospheres or nanocapsules that range in size from 100 to 1000 nm. Nanospheres form a dense matrix with dispersed actives or a matrix with surface-adhered actives, whereas nanocapsules act as a reservoir protecting the actives in the capsule interior or in their polymeric membrane [26, 27]. The shell formers poly lactic acid, polylactide-co-glycolide, and poly (Ɛ-caprolactone) are commonly used for these purposes. Polycaprolactone (PCL) is approved by the US-FDA and is used to prepare different nanocapsules [25]. These materials are interesting for topical applications because of their suspension stability and high surface area [26] and have been essential oil carriers [25, 30].

In addition, hydrogels are attractive delivery vehicles as they promote skin hydration and act as a protective barrier while facilitating repellant application. The use of nanoparticles incorporated into hydrogels (NP-gels) can improve mechanical characteristics of the bas hydrogels [31]. Hydrogels are 3D grids composed of hydrophobic or hydrophilic polymers that can act as nanoparticle carriers [32] while absorbing significant amounts of water, with similar characteristics as the extracellular matrix and other biocompatible materials [28–30].

Hydrogels can be physically (reversible) or chemically (irreversible) [33, 34] crosslinked, obtained from natural or synthetic [35] sources, and carry nanomaterials loaded with essential oils [36, 37].

**Fig. 1:**
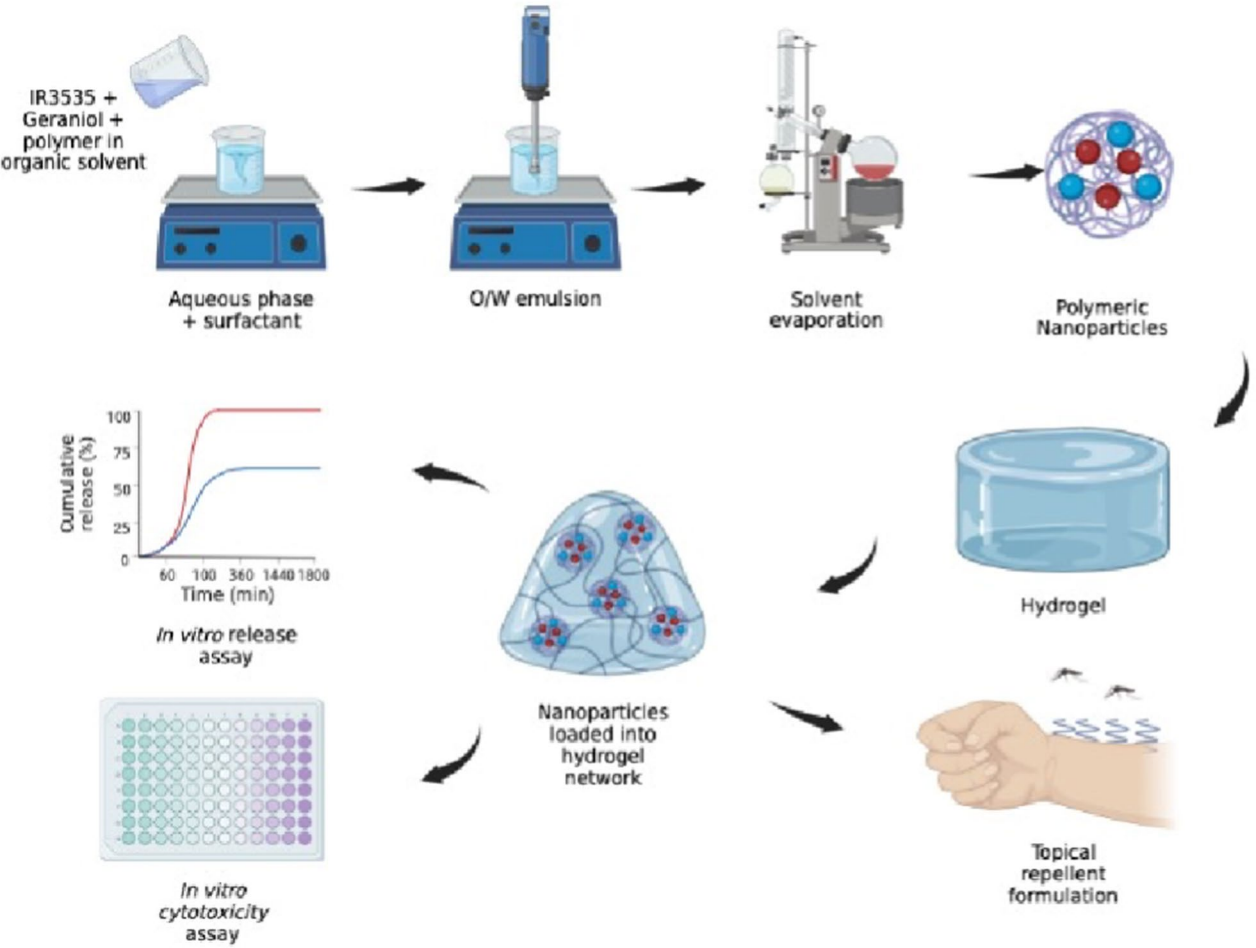
Schematic summary of the particle preparation, as well as the steps used in this study

Therefore, the aim of this study was to prepare and characterize a mixture of IR3535 and geraniol encapsulated into PCL nanoparticles using the emulsification/evaporation method followed by their dispersion into poloxamer-based hydrogels. The physicochemical characterization of nanoparticles in suspension as well as the rheological properties of the hydrogel containing nanoparticles were assessed. In addition, the toxicity was evaluated by MTT and disk diffusion assays, and the skin permeation studies and mechanism were evaluated using StratM® membranes and porcine ear, respectively (Fig. 1). This study aimed to provide potential alternatives for future application as repellent formulations to promote improved arboviruses control.

## Materials and methods

### Materials

Ethyl butyl acetylamine propionate butylacetylaminopropionate 98% (IR3535) and icaridin 97% (ICA) was obtained from Chemidin (China). Geraniol 98% (GRL) was obtained from Quinarí (Ponta Grossa, Paraná, Brazil). Tripalmitin, polyvinyl alcohol (PVA), and poloxamer 407 (PL407) were obtained from Sigma-Aldrich (St. Louis, USA). Mirytol was obtained from BASF. Ethanol was purchased from Labsynth (Diadema, São Paulo, Brazil). Acetonitrile (HPLC grade) was obtained from J.T. Baker (USA). All other reagents were obtained from local suppliers in Sorocaba (São Paulo, Brazil).

### Preparation of polymeric poli-(epsilon-caprolactone) nanoparticles

The nanoparticles were prepared using the emulsion/solvent evaporation method [38]. The organic phase was composed of mirytol, poli-(epsilon-caprolactone), and active agents (IR3535 and GRL) diluted in dichloromethane. This phase was mechanically mixed (Turrax homogenizer, 14,000 rpm) with the aqueous phase composed of 0.5% PVA (polyvinyl alcohol) surfactant. The solvent was evaporated until the formulation reached a final volume of 10 mL, resulting in active agent concentrations of 5 and 2% (w/v) for IR3535 and GRL, respectively. Empty PCLs were synthesized, and an emulsion formulation composed of bioactives in a 1.25% PVA solution were used as controls.

### Preparation of nanoparticle-poloxamer-based hydrogels

PCL nanoparticles were synthesized and incorporated into 30% (w/v) P407-based hydrogels. First, adequate amounts of P407 were solubilized in water in an ice bath for 12 h and maintained at 8 °C until use. PCL nanoparticle suspensions were incorporated into hydrogels via overnight homogenization at refrigerator temperature (4 °C).

### Physicochemical characterization of the PCL nanoparticles in suspension

The size distribution and polydispersity index (PDI) measurements were performed using Dynamic Light Scattering (DLS). Zeta potential determination was performed by microelectrophoresis using a ZetaSizer Nano ZS90 system (Malvern Instruments, UK) operated with a fixed angle of 90° at 25 °C with 200-fold diluted samples. In addition, nanoparticle concentration, size distribution, and polydispersity measurements were performed by nanoparticle tracking analysis using a NanoSight instrument (Malvern Instruments, UK) equipped with a green laser (532 nm) and ana sCMOS camera controlled by NanoSight v.3 software with the samples diluted 20,000 times. The results were obtained as the average of triplicate analyses. The formulations were maintained at room temperature (25 °C) and their stability was evaluated over time with measurements taken at 0, 15, 30, 60, 90, and 120 days. The encapsulation efficiency was determined using microcentrifugation, with quantification of the non-encapsulated active compounds in the filtrate by high-performance liquid chromatography (HPLC) using an Ultimate 3000 instrument (Thermo Fisher Scientific, Waltham, USA) with Chromeleon 7.2 software for chromatogram acquisition and analysis. The mobile phases were methanol:water (65:35 v/v) and acetonitrile: water (60:40 v/v) with a Phenomenex Gemini C18 column (150 × 4.60 mm, 5 µm). All tests were performed in triplicate. The calculations were based on the GRL and IR3535 calibration curves with the equations y = 1.28395x + 2.60255 (R^2^ = 0.99894) and y = 0.52575.x – 0.06279 (R^2^ = 0.9997), respectively.

### Repellent formulation morphology using atomic force microscopy

The nanoparticle morphology and size distributions were analyzed by atomic force microscopy (AFM) using a Nanosurf Easyscan 2 Basic BT02217 instrument. The nanoparticle suspensions were diluted 20,000 times, dispersed on silicon plates, and dried in a desiccator. For nanoparticles in the gel, a smear of the formulation was used without dilution. The images (256 × 256 pixels, TIFF format) were obtained using a TapA1-G probe (BudgetSensors, Izgrev, Bulgaria) in tapping mode at 90 Hz. The images were processed using Gwyddion software (Dublin, Ireland). All morphological analyzes were performed for both nanoparticle suspensions and hydrogel formulations.

### Hydrogel mechanical properties: rheological analysis under frequency, temperature, and shear stress variation

Rheological studies were performed to analyze the formulation mechanical behavior under temperature, frequency, and shear stress variations to examine the system structural organization and stability. Hydrogel rheological properties under temperature and frequency sweeps were analyzed using an oscillatory rheometer (Kinexus lab, Malvern Instruments, Malvern, UK) with cone and plate geometry (20 mm diameter, 0.5 rad angle, and 1 mm gap). The repellent formulations (1 g) were placed in the sample holder and evaluated at skin temperature (32.0 ± 0.5 °C). The first analysis was performed under a temperature ramp from 10 to 50 °C at 5 °C/min and 1 Hz frequency and 1 Pa shear stress. In addition, oscillatory analyses were performed under frequency sweep from 0.1 to 10 Hz with a shear stress of 1 Pa at 32.5 °C.

In another approach, continuous rheological measurements were performed using a plate-plate geometry and 0.1 mm gap. The elastic Gʹ and viscous moduli G" were measured between 0 and 60 °C. Gʹ and G" measurements were also made with ω frequency from 0.1 to 10 Hz at 32.5 °C [39]. The flow curves σ (γ ˙) (“backward” and “forward”) were obtained at 32.5 °C with a shear rate γ ˙ ranging from 0 to 100 s^−1^. Self-healing curves were obtained at 32.5 °C, frequency of 1 Hz, varying the rate by 1% and 100% with equal time intervals.

With the G' data as a function of the frequency ω (in Hz) the adhesion parameter S was calculated from the following relationship (Eq. 1).

1 $${\text{G}}^{\prime} = {\text{S}} \cdot {\upomega} (\text{n})$$

where n is an adimentional parameter that indicates material structuring. The calculation of S is related to material cohesion on a smooth surface, being proportional to the work required to remove it from that surface [40, 41] and is calculated as follows (Eq. (2)).

2 $${\upsigma} = {\text{K}} \cdot {\text{y}}({\text{m}})$$

The consistency (K) and viscosity index (m) values are calculated from the flux curve data of σ (γ ˙) in both directions. K is defined as a mechanical property, proportional to material stiffness, while the m index allows comparison of spreadability of each material [40, 41].

### Bioactive release profiles and mechanisms under in vitro release and permeation conditions

Investigation of the in vitro release profiles for the liquid and gel PCL formulations employed a vertical diffusion system with a regenerated cellulose membrane (MWCO 12,000–14,000 pores) coupled to donor and receptor compartments. The system was operated under sink conditions. First, 100 mL of 3% PVA was used as co-eluent in the receptor solution and the system was kept in a closed environment at 32.5 °C under constant magnetic stirring [42]. Formulations were applied (1 g of gel or 1 mL of suspension) in the donor compartment, after which 1 mL aliquots were periodically removed from the receptor compartment, replacing the volume removed by co-eluent solution. Geraniol and IR3535 were quantified via HPLC methods, as described above. The release profile data were analyzed considering different mathematical models (zero-order, first-order, Higuchi, and Korsmeyer-Peppas) to investigate the release mechanisms of the active agents from the different systems.

Permeation kinetic assays were performed in a two-compartment vertical diffusion cell system (Microette Plus, Hanson Research, Chatsworth, CA, USA) with donor (1.72 cm^2^ permeation area) and receptor compartments (7 mL) separated by a synthetic membrane (Strat-M®, 25 mm diameter, Millipore Co., USA) [43, 44].

Sample formulations (0.6 g) were placed in the donor compartment in contact with the membrane and the receptor compartment was filled with 0.1 M sodium phosphate buffer (pH 7.4). The system was kept under magnetic stirring (350 rpm) at 32.5 ± 0.5 °C. Aliquots were withdrawn at 0, 0.5, 1, 2, 4, 6, 8, 12, and 24 h with bioactives quantified by HPLC. All formulations were evaluated in triplicate. The cumulative amount of repellent permeated, Q (mg/cm^2^) the Strat-M per unit area was plotted as a function of time (min). For data analysis, the flux values were obtained from the slope of the concentration versus time curve in the linear portion (interval between 15 and 450 min). The data were analyzed according to Eq. (3).

3 $${\text{J}} = {\text{P}} \times {\text{Cd}}$$

where J (% cm^−2^ min^−1^) is the compound flux through the membrane, P (cm min^−1^) is the permeability coefficient, and Cd (% cm^−3^) is the compound concentration in the donor compartment [45]. Lag time (t_lag_) was calculated from the intercept (Y = 0).

### Permeation mechanisms: epidermis structural analysis

Porcine ear samples were obtained from a local slaughterhouse and the experimental protocol was approved by the Federal University of ABC—Institutional Committee for the Care and Use of Animals (#8719010318). Blood vessels and subcutaneous tissue were removed and skin dermatomed at 0.45 mm (Nouvag, Rorschach, Switzerland). For epidermis separation, the skin samples were immersed in a water bath at 60 °C for 3 min and the isolated epidermis was subsequently detached. Epidermis samples were stored at − 20 °C for a maximum of 3 months. For structural analysis, the epidermis surface (0.8 cm diameter) was treated with repellent formulations and kept in contact for 24 h at room temperature [39]. Formulations were then removed from the epidermis samples and analyzed by FTIR. FTIR spectra were obtained for the isolated formulation components, repellent formulations, while non-treated epidermis samples (0.8 cm diameter) were used as controls. FTIR spectra were recorded at 4000–650 cm^−1^ with 1 cm.^−1^ resolution using a PerkinElmer Spectrum Two 160.000A in ATR mode [42–44].

### In vitro cell viability assay

#### Cell viability based on mitochondrial activity

The 3-(4,5-dimethylthiazol-2-yl)-2,5-diphenyltetrazolium bromide (MTT) assay was used to determine the cytotoxic effects of the repellent formulations based on PCL nanoparticles in keratinocyte cell lines (HaCat) and fibroblasts (NIH/3T3). The cells were plated at 7000 and 8000 cells/well and incubated in a climate-controlled oven at 37 °C and 5% CO_2_. The cells were exposed for 24 h to either non-encapsulated or encapsulated repellents at 8 concentrations. The doses used for each activity ranged from 0.02 to 200 mg/mL for GRL and 0.05 to 500 mg/mL for IR3535. The control nanoparticles (empty) were tested at the same dilution. After 24 h of exposure, the cells were incubated with 0.5 mg/mL MTT for 4 h. Subsequently, the supernatants were removed and DMSO was added and incubated for 10 min at 37 °C to dissolve the formazan crystals and their absorbance was measured at 540 nm using a microplate reader. In living cells, mitochondrial dehydrogenase oxidizes MTT to formazan, while damaged or dead cells show reduced or no dehydrogenase activity. Data were reported as means of two independent experiments (three replicates each) ± standard error of mean and were expressed as percent viability with respect to the control.

#### Disk diffusion assay

Cells were plated in 60 mm Petri dishes at 1.5 × 10^5^ cells/mL using DMEM High culture medium with 10% fetal bovine serum and were grown for 48 h at 37 °C in an oven with 5% CO_2_. Then the liquid medium was discarded and the solid overlay medium (2 × concentrated Eagle medium, containing 1.8% agar and 0.01% neutral red as a vital dye) was added over the cell mat. After solidification, the test material was deposited in the center of the plate and incubated for 24 h at 37 °C in a 5% CO_2_ environment. The inoculated plate readings were performed macroscopically, where cytotoxicity was confirmed by the presence of a clear halo around the material, corresponding to dead cells and microscopically for morphological alterations of the cells surrounding the sample.

### Statistical analyses

The data were presented as mean ± standard deviation (SD). The Shapiro–Wilk test was used for evaluation of normality, followed by application of the two-way ANOVA test for multiple variables considering a significance level of 0.05. The software used was GraphPad Prism 8.0.1.

## Results and discussions

### Nanoparticle physicochemical characterization

**Fig. 2:**
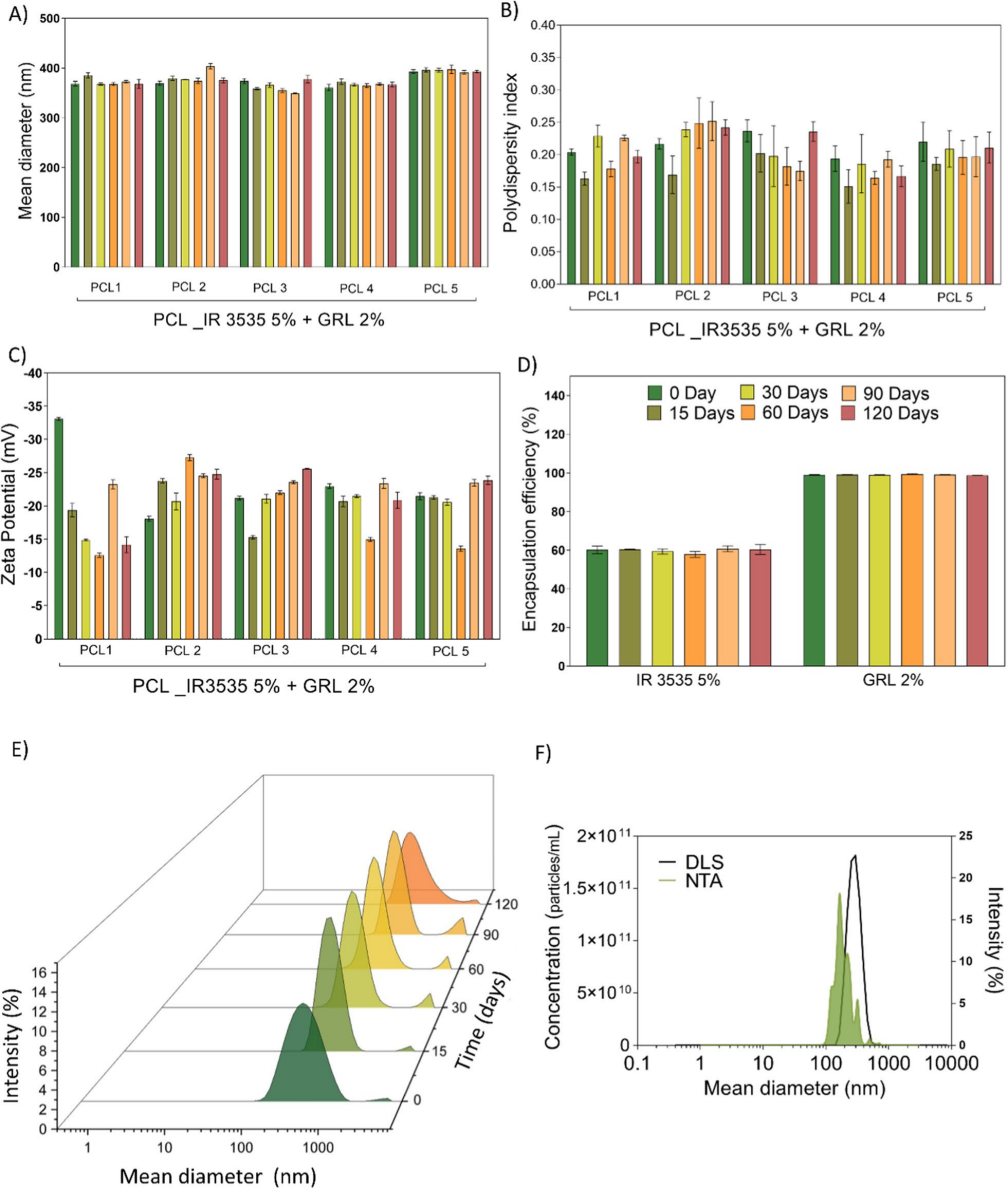
Analysis of the properties of PCLs along with the times 0, 15, 30, 60, 90, and 120 days for size (nm, **A**, and **B**); zeta potential (mV, **C**). The encapsulation efficiency (%, **D**) was expressed as the mean and standard deviation of the five batches of PCL formulation. **E** Distribution as a function of intensity (DLS) over 120 days; **F** Comparison between the diameter distribution as a function of concentration (NTA) and intensity (DLS). The respective values were expressed as mean and standard deviation (n = 3) for five identical PCL formulation lots loaded with 5% IR3535 and 2% geraniol (GRL)

The mean diameters (nm) of the five batches of identical PCL formulations encapsulated with 5% IR3535 + 2% geraniol were 373 ± 11, 378 ± 13, 374 ± 12, 371 ± 15, 377 ± 21, and 376 ± 5 nm (Fig. 2A). The samples passed the Shapiro–Wilk normality test, and the analysis of variance (ANOVA) did not indicate significant differences in particle size. The PDI and PZ were analyzed in the same manner where the PDI values over 120 days were 0.21 ± 0.01, 0.17 ± 0.02, 0.21 ± 0.02, 0.19 ± 0.03, 0.20 ± 0.03, and 0.21 ± 0.01(Fig. 2B).

PDI values of > 0.2 are commonly deemed acceptable in practice for polymer-based nanoparticles in agreement with the DLS size distribution analysis over time shown in Fig. 2E. The PZ values were − 23 ± 5, − 20 ± 2, − 19 ± 2, − 18 ± 6, − 23 ± 0.5, and − 21 ± 0.7 mV with no significant differences as a function of time. The encapsulation efficiencies (EEs) of the active compounds IR3535 and GRL were evaluated over 0.15, 30, 60, 90 and 120 days showing 60.1 ± 1 0.9%, 60.4 ± 0.1%, 59.3 ± 1.3%, 57.8 ± 1.4%, 60.7 ± 1.5%, and 60.2 ± 2%, respectively, for IR3535. For geraniol, EE values were 99 ± 0.1%, 99 ± 0.03%, 99 ± 0.09%, 99 ± 0.3%, 99 ± 0.1%, and 99 ± 0.2%, respectively for each time point (Fig. 2A–D). Similarly, in a sunscreen formulation with PCL, the average nanoparticle size was determined to be 418 ± 56 and with a PDI of 0.36 ± 0.062 [49]. In addition, roxithromycin was encapsulated in PCL nanoparticles for hair follicle treatment, yielding a mean diameter of 300 nm and maximum EE of 61% [50]. Therefore, the evaluated parameters obtained herein agree with studies performed using similar systems.

The AFM size distribution histogram (Additional file 1: Fig. S1A) showed mean diameters between 200 and 800 nm (analysis of 100 particles using the Gwyddion software). In contrast, the average size of the PCLs was determined to be approximately 400 nm by DLS. The sample PDI which was evident in the size distribution determined by DLS (Fig. 2E) and the aggregation of nanoparticles during the drying process (ubiquitous in morphological studies) explain this variation [51]. In addition, the topographic images demonstrate a spherical morphology, which is a characteristic related to the efficiency of nanoparticle synthesis. The surface topography of hydrogels containing PCL nanoparticles loading IR3535 and GRL and subsequently dried was determined by AFM. According to the topographic and 3D image (Additional file 1: Fig. S1D, E) it is possible to see areas with with high variability of protusions (height) and rough surface of the bydrogel matrix. In addition, it was possible to see the nanoparticles embedded into hydrogel matrix. In addition, through the images it is possible to see the presence of nanoparticles embedded into hydrogel matrix.

### Hydrogel rheological analysis

The incorporation of PCL nanoparticles or emulsions into hydrogel formulations tends to form structurally organized systems, as observed by the increased Gʹ and viscosity values. Although emulsion-gel and PCL-hydrogel viscosities showed values close to 4.5 ∙ 10^6^ mPa s, the emulsion-gel was the most structured material, as evidenced by the Gʹ/G'' ratio of 351.9 (Table 1). Both emulsion-gel and PCL-gel formulations were stable between 20 and 40 °C and the incorporation of additives (emulsion and nanoparticles) decreased the sol–gel transition temperature for all systems.

**Table 1:** Rheological parameters of control emulsion, control emulsion incorporated into the hydrogel (emulsion-hydrogel), PCL nanoparticles (PCL) and PCL nanoparticles incorporated into hydrogels (PCL-hydrogel) measured at 32.5 ºC

Formulation	Gʹ/G" (32.5 °C)	η* (mPa.s) (32.5 °C)	S	n	R^2^	K
Emulsion	8.7 ± 0.8	40.6 ± 5.1	0.21 ± 0.01	2.12 ± 0.02	0.99	0.0597 ± 0.001
Emulsion-hydrogel	351.9 ± 7.1	4.2 ± 0.1 (.10^6^)	14,266 ± 80	0.079 ± 0.004	0.95	745.8 ± 9.8
PCL	0.40 ± 0.15	226.6 ± 25.0	80.7 ± 1.3	0.39 ± 0.01	0.99	0.015 ± 0.002
PCL-hydrogel	52.3 ± 1.6	4.6 ± 0.9 (.10^6^)	19,354 ± 261	0.07 ± 0.01	0.73	619.5 ± 23.0

Gʹ/G"—elastic x viscous moduli relationship, η*—viscosity; S—adhesion, n—flow index, K—consistency index, R^2^—linearity coefficient

**Fig. 3:**
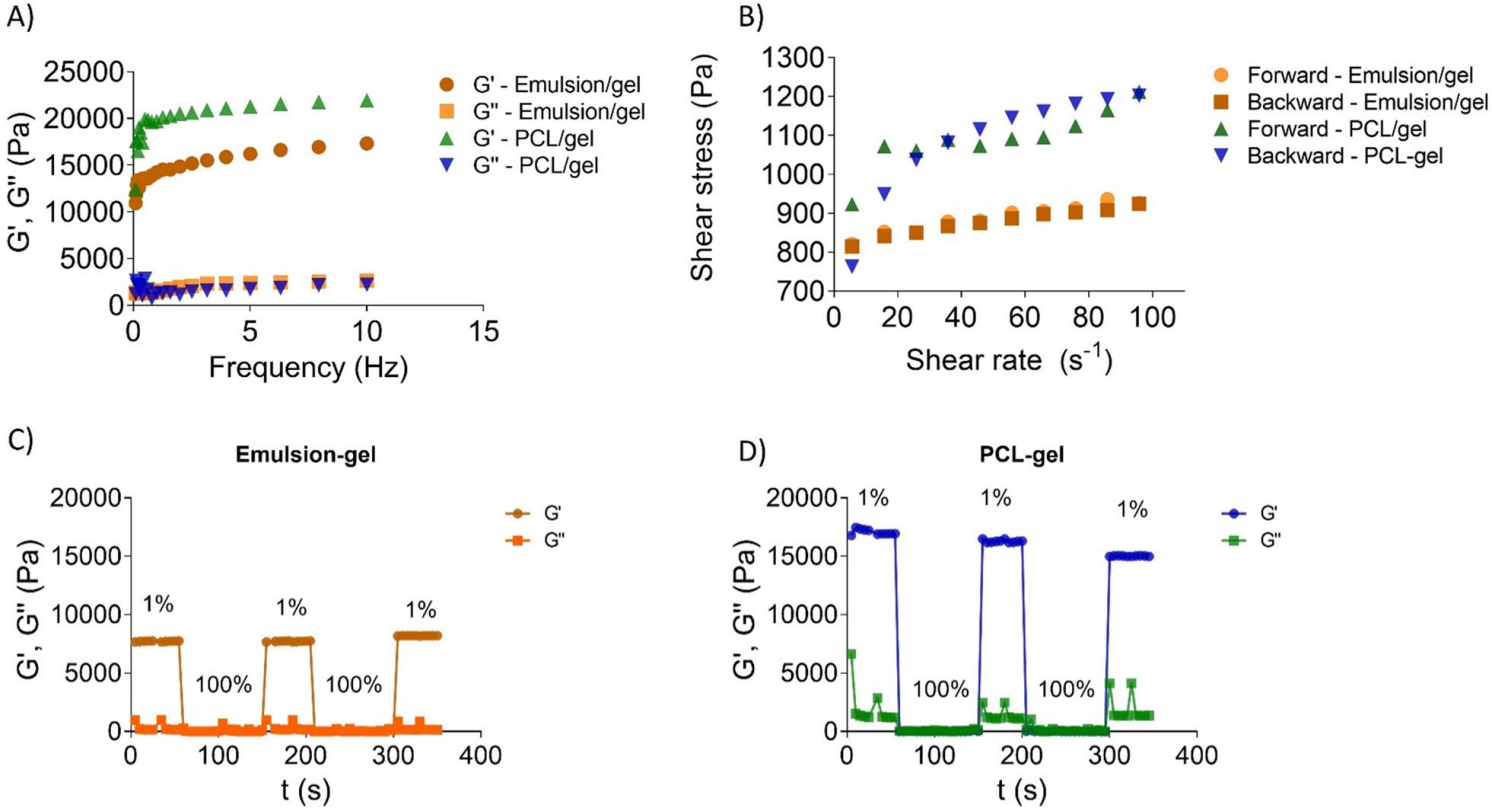
Rheological characterization of poloxamer hydrogels (30% w/v) containing non-encapsulated IR3535 nd GRL and hydrogel containing PCL nanoparticles loading IR3535 nd GRL. **A** Rheological properties as function of frequency sweep analysis (0.1–10 Hz) at 32.5 ºC; **B** flow curves expressed as shear rate (0–100 s^−1^) and applied shear stress; **C** Self-healins profiles obtained at 1 Hz and at 32.5 ºC for non-encapsulated IR3535 and GRL in hydrogels and **D** PCL nanoparticles loading IR3535 and GRL incorporated in hydrogels

Frequency sweep analysis allowed materials stability determination when exposed to oscillatory movement. This movement increased PCL-hydrogel stiffness compared to the emulsion-hydrogel. In addition, PCL hydrogels showed greater stability over the tested frequency range compared to the other systems (Fig. 3A). Thus, it can be inferred that the PCL-hydrogel exhibited greater cohesion between the formulation components, as indicated by the high adhesion value (S) compared to those obtained for the other formulations (Table 1).

**Fig. 4:**
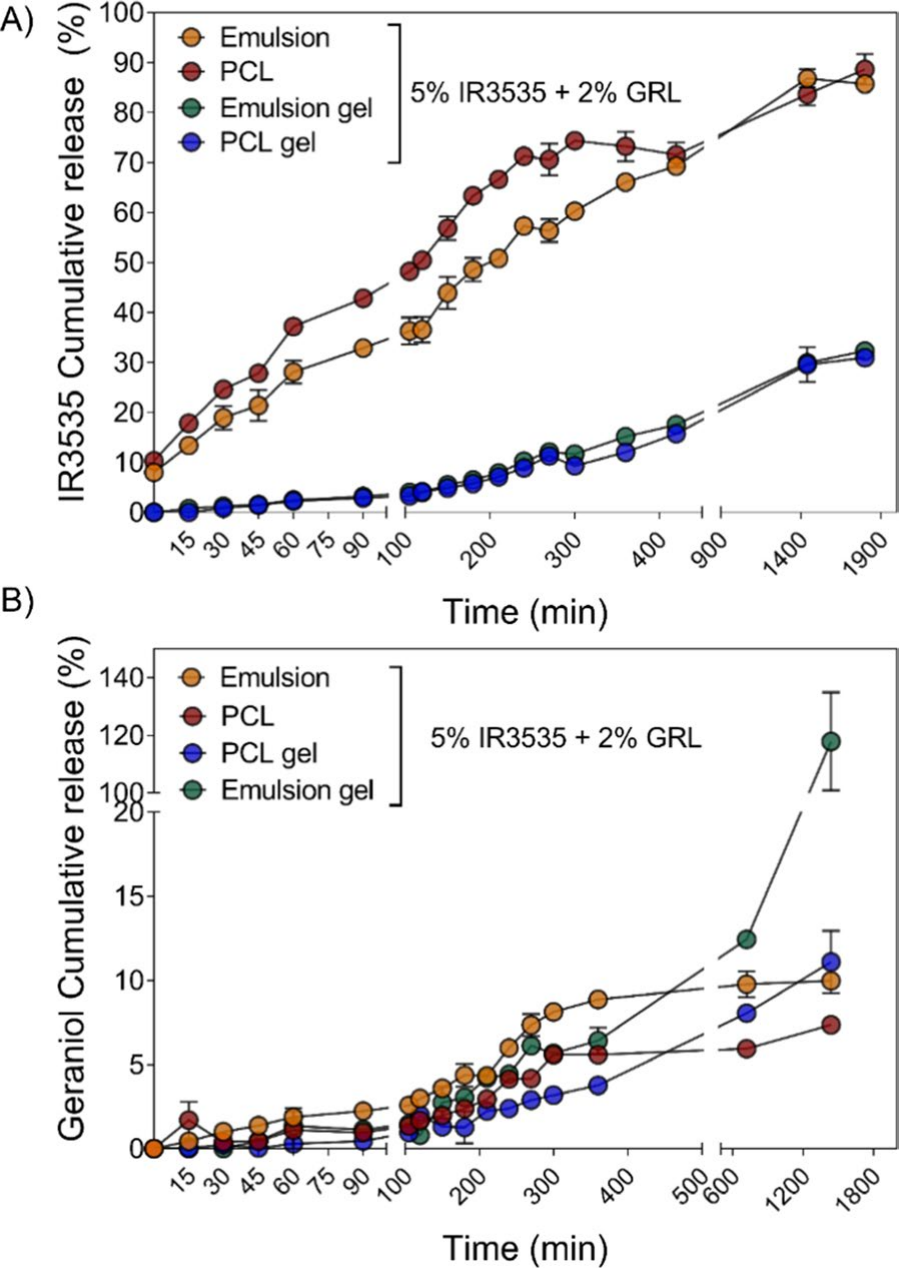
In vitro release assay of IR3535 and GRL from PCL nanoparticles and PCL nanoparticles incorporated into hydrogels. The release assay was performed using the vertical diffusion cell system at 32.5 °C, in aqueous media containing 3% of PVA as co-eluent over 30 h. Values represent the mean ± SD (n = 3)

As the PCL-hydrogel exhibited greater structural cohesion, this formulation is expected to exert greater flow resistivity than the emulsion-hydrogel. The frequency rheological results and flow curves showed that the PCL- and emulsion-gels were more responsive to movement than the emulsion and PCL alone, since the gel affected the molecular organization of both formulations. The viscosity index (m; Table 1 and Fig. 3B) showed that the emulsion and PCL alone exhibited more of a liquid character than the gel formulations, considered to be pseudoplastic behavior. This pseudoplastic character shows that the gel reduces materials outflow, a favorable indicator for topical applications [39, 40, 52, 53] as repellents must remain on the skin surface at the application site.

The consistency values (K) indicate that incorporation into the hydrogel increased the material stiffness. The emulsion-gel showed greater consistency than the PCL-hydrogel, confirming a greater elastic than viscous character when the emulsion is incorporated into the hydrogel. In fact, emulsion or PCL nanoparticle addition allowed structural recovery even under shear stress variation, as evidenced by the self-healing experiments results (Fig. 3C, D). As shown by the flow curve of the PCL-gel, disruption occurs when subjected to voltage oscillation, decreasing the elastic modulus Gʹ over time. In contrast, the emulsion-gel is more stable under stress variation, with Gʹ values approximately the same as that at 1% shear stress.

### Repellent release profiles and mechanisms under in vitro release and permeation conditions

Nanoparticle suspensions and hydrogel formulations containing nanoparticles showed a sustained release profile for both IR3535 and GRL bioactives. A significant (p < 0.05) increase in IR3535 release from PCL nanoparticles was observed up to 6 h compared to IR3535 in the emulsion (liquid), whereas the concentrations released were similar after 24 h. However, the suspensions incorporated into poloxamer hydrogels showed no significant differences in release rates between IR3535 encapsulated in nanoparticles (PCL gel) and emulsified with PVA (Emulsion_gel). However, the GRL release profile from the emulsion formulation (Emulsion_liq.) showed significantly (p < 0.05) higher concentrations than the encapsulated GRL in nanoparticles (PCL liq.). Additionally, no significant differences in the release profiles between the emulsion and NPs over 12 h were observed after the suspensions were incorporated into poloxamer hydrogels. Although, a considerable increase in GRL was released from the emulsion_gel after 24 h (Fig. 4B).

The hybrid systems (nanoparticles + hydrogel) showed a gradual IR3535 release profile compared to the nanoparticle suspension (PCL liq.) under the same release conditions. In contrast, there was significant increase in GRL release rate after 4 h (210 min, p < 0.05) in a hybrid system (PCL gel) compared to the nanoparticle suspension (PCL liq.). Corroborating these results, Nnamani et al. [54] reported that the release rate of artemether (ART), a malaria therapeutic, decreased after its incorporation into a hybrid system (NLC + Poloxamer 407) compared to encapsulation in liquid NLC. PCL encapsulated GRL and IR3535 released 7.3 ± 0.1 and 83.6 ± 2.2% at 24 h, respectively. The same formulation incorporated into a poloxamer 407 hydrogel released 11 ± 0.9% and 29.5 ± 3% of GRL and IR3535, respectively. All formulations demonstrated a sustained release pattern, indicating a high rate of polymer solubilization and hydration in the release medium. The faster release rate of IR3535 in both formulations when compared to the release rate of GRL, could be related to the difference in aqueous solubility, which is around 700 times higher to IR3535 (70 g/L) when compared to GRL (0.1 g/L). Due to the higher solubility of IR3535, the molecules are more likely located near to the nanoparticles surface when compared to GRL and consequently, the release rate is faster [55, 56].

Additional file 1: Table S1 shows the data from the release kinetic models applied to the liquid and gel PCL formulations. Based on the linear coefficient (R^2^) values, the predominant GRL release model in the liquid and gel formulations was zero-order kinetics, suggesting a prolonged release model. The Higuchi model best described the release of IR3535 in the liquid formulation, indicating that diffusion was the predominant mechanism of drug release from the nanoparticles to the receptor medium. In the gel formulation, the Korsmeyer-Peppas model was a better fit to IR3535 release, with the values of the release exponent “n” of > 1. This is characteristic of case II transport, showing the contribution of both drug diffusion and hydrogel erosion caused by polymeric chains hydration and relaxation [57].

Through the application of mathematical models in the release profile, it was possible to observe that the release of IR3535 is mainly governed by the diffusion of the molecule to the medium in both formulations, although there was a difference in the model that explains the release. On the other hand, the GRL release mechanisms fit the zero-order model regardless of the formulation used. This model indicates that the release rate of the molecule is constant over a period. In this way, it is believed that the faster release of IR3535 will guarantee the most immediate repellent effect and the slower and sustained release of geraniol will be able to guarantee the long-term repellency. Thus, the molecules will guarantee the synergistic and complementary insect repellent effect. These results are in agreement with previous reports related to polymeric nanocapsules and poloxamer 407 hydrogels [27, 58].

The release kinetics allowed examination of formulation performance as drug release matrices. According to the models, different release profiles between the GRL and IR3535 were observed. This combination is interesting and open perspectives for developing a repellent formulation with prolonged action and a possible synergistic effect promoted by the mixed mechanisms of the components.

**Fig. 5:**
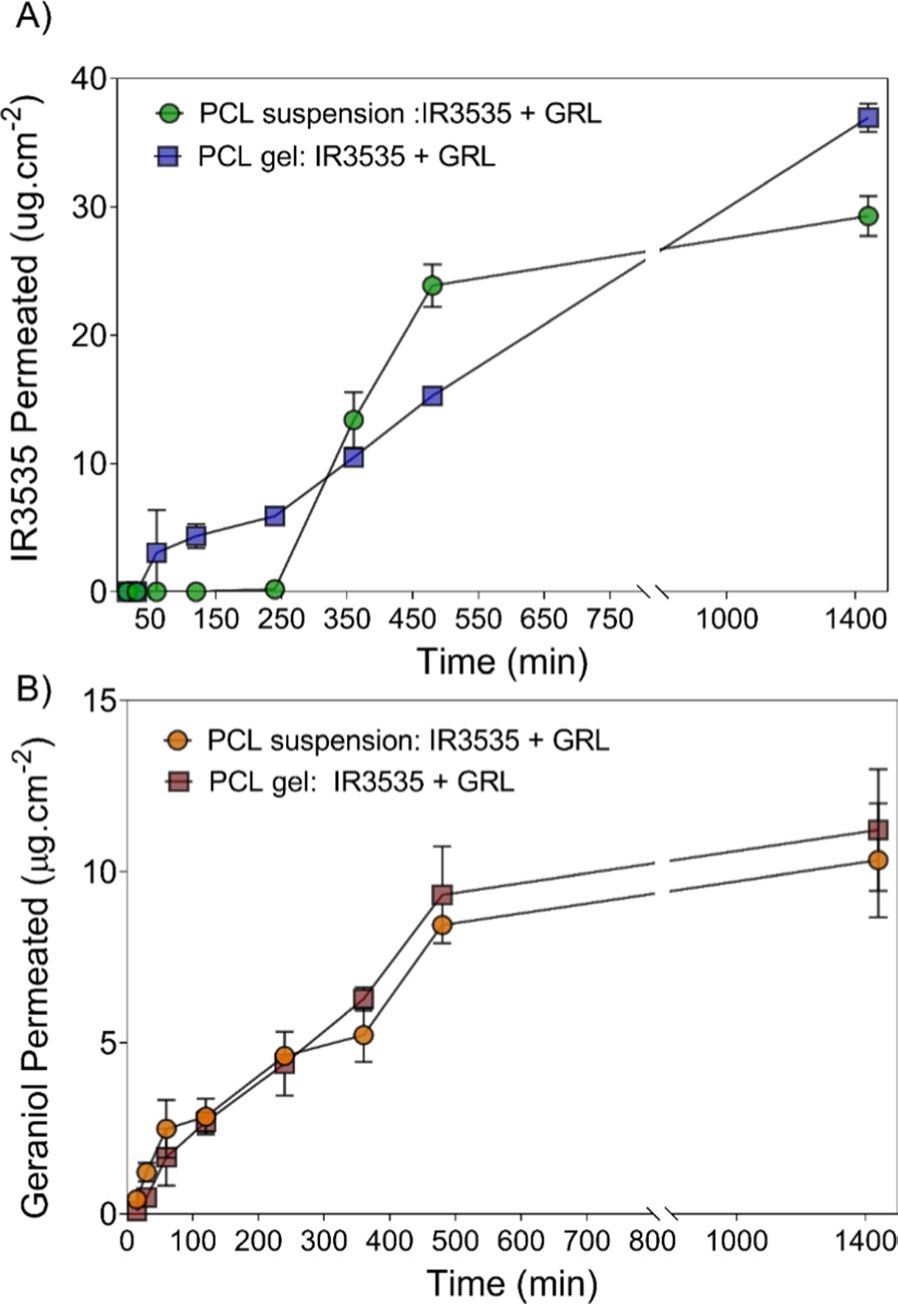
In vitro permeation profile of PCL nanoparticles suspension and PCL nanoparticles incorporated into hydrogels. The permeation study was performed using vertical diffusion cell at 32.5 ºC, in phosphate buffer pH 7.4. **A** IR3535 permeation profile and **B** GRL permeation profile. The values represent the mean ± SD (n = 3)

In vitro permeation assays were also performed to predict bioactives permeation profiles from the nanoparticles and hydrogels as well as their performance when in contact with the skin surface (Fig. 5). According to analyzed data, for all formulations the repellent concentration released as a function of time showed a linear relationship, as confirmed by the correlation coefficients (R^2^ = 0.926–0.981), except for the IR3535 released from the liquid PCL formulation, which showed a burst release after 6 h. Both GRL and IR3535 permeation results showed differential effects depending on formulation type, nanoparticle suspension or hydrogel. GRL permeation from PCL nanoparticles and PCL nanoparticles in the hydrogel showed similar profiles, where high GRL permeated concentrations were observed after 8 and 24 h. The GRL loading into the PCL and PCL-hydrogel was 20 mg/g, indicating that < 1% permeated after 24 h. This showed that both formulations (PCL nanoparticles and PCL in the hydrogel) controlled bioactive percutaneous absorption. GRL flux values were 0.015 and 0.019 µg cm^−2^ min^−1^ for the PCL nanoparticles and PCL in hydrogels, respectively (Table 2). In contrast, IR3535 permeation was modulated by the formulations, where permeated amounts were higher for the PCL nanoparticles in hydrogels (36.9 ± 1.1 mg/cm^2^) compared to IR3535-PCL nanoparticles (29.2 ± 1.5 mg/cm^2^). These profiles exhibited flux values of 0.023 and 0.026 µg cm^−2^ min^−1^ for the PCL nanoparticles and PCL in hydrogels, respectively. However, the most pronounced difference was observed during initial permeation time, with values of ~ 250 and 50 min observed for PCL nanoparticles and PCL nanoparticles in hydrogels, respectively (Table 2).

**Table 2:** Permeation parameters of IR3535 and GRL across Strat-M® artificial membranes from PCL nanoparticles loading both compounds (GRL + IR3535) and PCL nanoparticles loading both compounds (GRL + IR3535) incorporated into hydrogel

Formulations	Flux (10^–4^ mg cm^−2^ min^−1^)	Permeability coefficient (10^–4^ cm min^−1^)	Lag time (min)
*PCL suspension*			
GRL	148.2	74.1	57.2
IR3535	227.0	45.4	5.62
*PCL gel*			
GRL	185.3	92.6	24.2
IR3535	257.3	51.4	26.1

Similar results were reported in other studies regarding the permeation of ICA and GRL from NLC gel formulations. A 14-fold increase in permeation of ICA and 40-fold increase of GRL from NLC incorporated in the HPMC gel were reported [59]. Comparing NLC and PCL formulations, the permeation rate of synthetic actives (ICA and IR3535) through the Strat-M membrane was higher for IR3535 compared to the liquid formulations. However, comparing NLC and PCL in gels, the permeation rate of ICA was 6 times higher. The lag times (T_lag_) of synthetic actives were similar (23.8–26.1 min), but the natural active GRL showed a significant difference in NLC and PCL suspension at 591.4 and 57.2 min, respectively. Similarly, comparing the NLC and PCL gels, significant differences were observed at 43.9 and 5.6 min, respectively. Briefly, the NLC suspension showed a tenfold increased T_lag_ compared to PCL in the suspension formulation and the NLC gel exhibited an eightfold increased T_lag_ compared to the PCL gel.

### Permeation mechanisms: epidermis structural analysis

The literature reports a natural repellent effect in human skin originating from the unsaturated fatty acids present in stratum corneum (SC) lipid matrix [60]. For repellent topical applications, protection depends mainly on formulation retention on the skin surface, avoiding permeation of the bioactive [61]. Thus, preservation of the stratum corneum barrier integrity after using repellent formulations is necessary. FTIR was used to evaluate the interaction between the gels and SC after 4 h of contact.

According to Boncheva et al., CH_2_ stretching vibrations are powerful markers to indicate structural changes in the SC lipid matrix. Parameters including the shape of the CH_2_ stretching bands and its precise wavenumber are related to lipid supramolecular organization in the SC. When the −CH_2_ symmetric stretching band is centered at a wavenumber of < 2850 cm^−1^ the lipids are organized in an orthorhombic (OR) structure. At > 2850 cm^−1^ the lipids transition from OR to hexagonal (HEX) or liquid-crystalline (LIQ) phases [46]. In porcine ear, the SC lipid organization is characterized by mixed OR and HEX phases [47, 48].

Herein, symmetric CH_2_ stretching for the control sample was observed at 2851 cm^−1^, confirming a mixture of OR and HEX phases. The treated samples showed lower CH_2_ stretching band resolution due to overlap of the CH_2_ symmetric and asymmetric stretching bands. Consequently, enlargement of the CH_2_ stretching symmetric band reflected structural changes in the SC lipid matrix organization.

Previous studies showed a high permeation capacity for repellent molecules regularly used in commercial formulations [62], e.g., DEET. Besides being considered safe for dermal applications, extensive literature discussions regarding the correlation between exposure to DEET and negative health effects are ongoing [63]. In contrast, GRL is a natural control agent exhibiting low toxicity in mammals [19, 64] and IR3535 is a derivative of β-alanine with very low toxicity and similar efficacy as DEET [65].

**Fig. 6:**
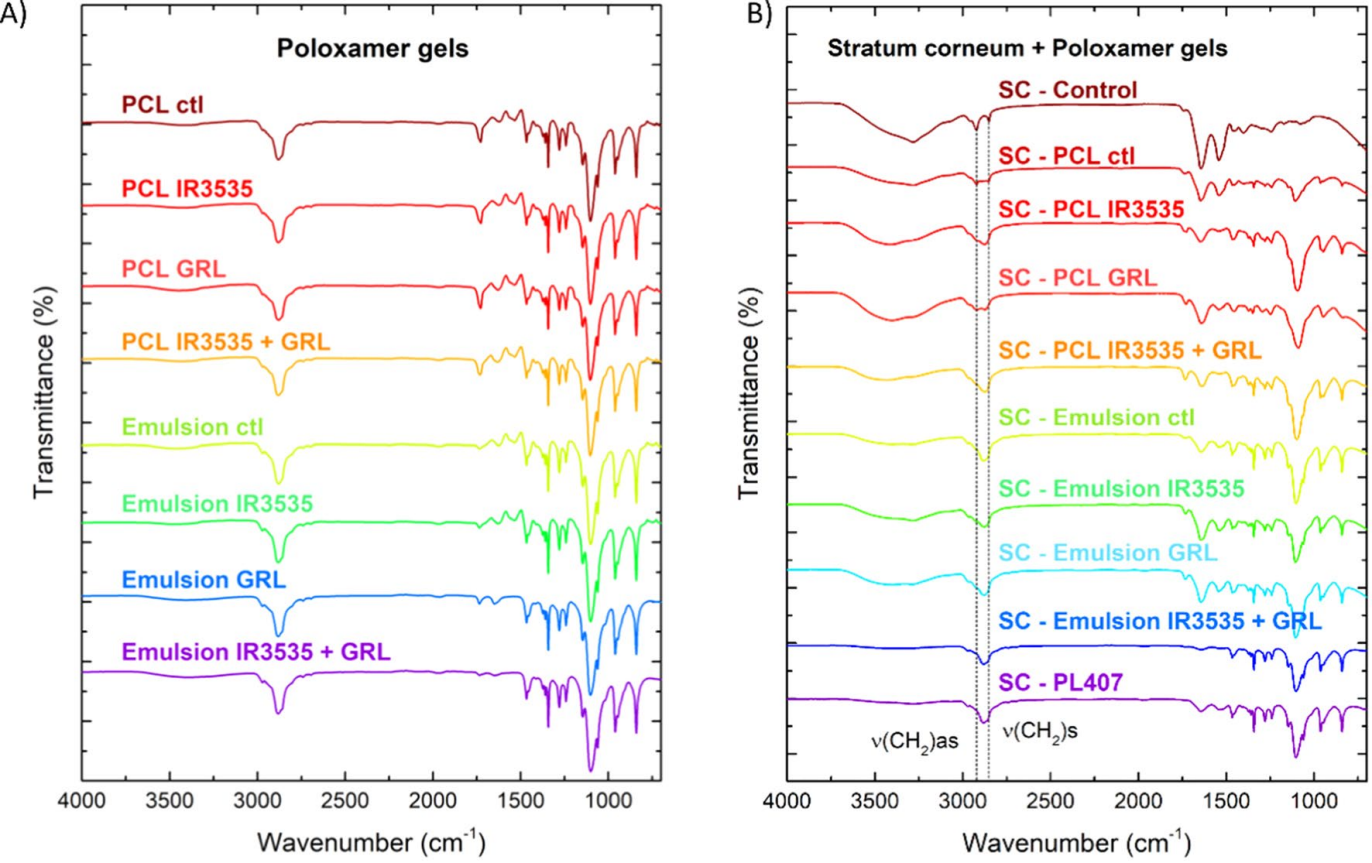
Infrared characterization of **A** poloxamer gels and additives, and **B** stratum corneum samples after treatment with the gels per 4 h. FTIR spectra were recorded in the 4000–650 cm^−1^ range with 1 cm^−1^ resolution. The treatments used were: PCL nanoparticles (PCL ctl), PCL nanoparticles loading IR3535 (PCL IR3535), PCL nanoparticles loading GRL (PCL GRL), PCL nanoparticles loading both compounds (PCL IR3535 + GRL), control emulsion, non-encapsulated IR3535 (Emulsion IR3535), non-encapsulated GRL (emulsion GRL) and mixture of non-encapsulated compounds (Emulsion IR3535 + GRL)

As a monoterpene, GRL has been applied as a natural additive to improve drug skin penetration in formulations containing caffeine and diclofenac sodium. Thus, interactions between the formulations and SC were expected for samples containing GRL [19]. Permeation analysis (Fig. 6) showed limited skin permeation behavior for gel formulations compared to the synthetic molecules [62]. Therefore, SC changes caused by interactions with formulations may not represent a safety risk to consumers, but the product efficacy can be impaired due to the repellent permeation [62].

### In vitro cell viability assay

**Fig. 7:**
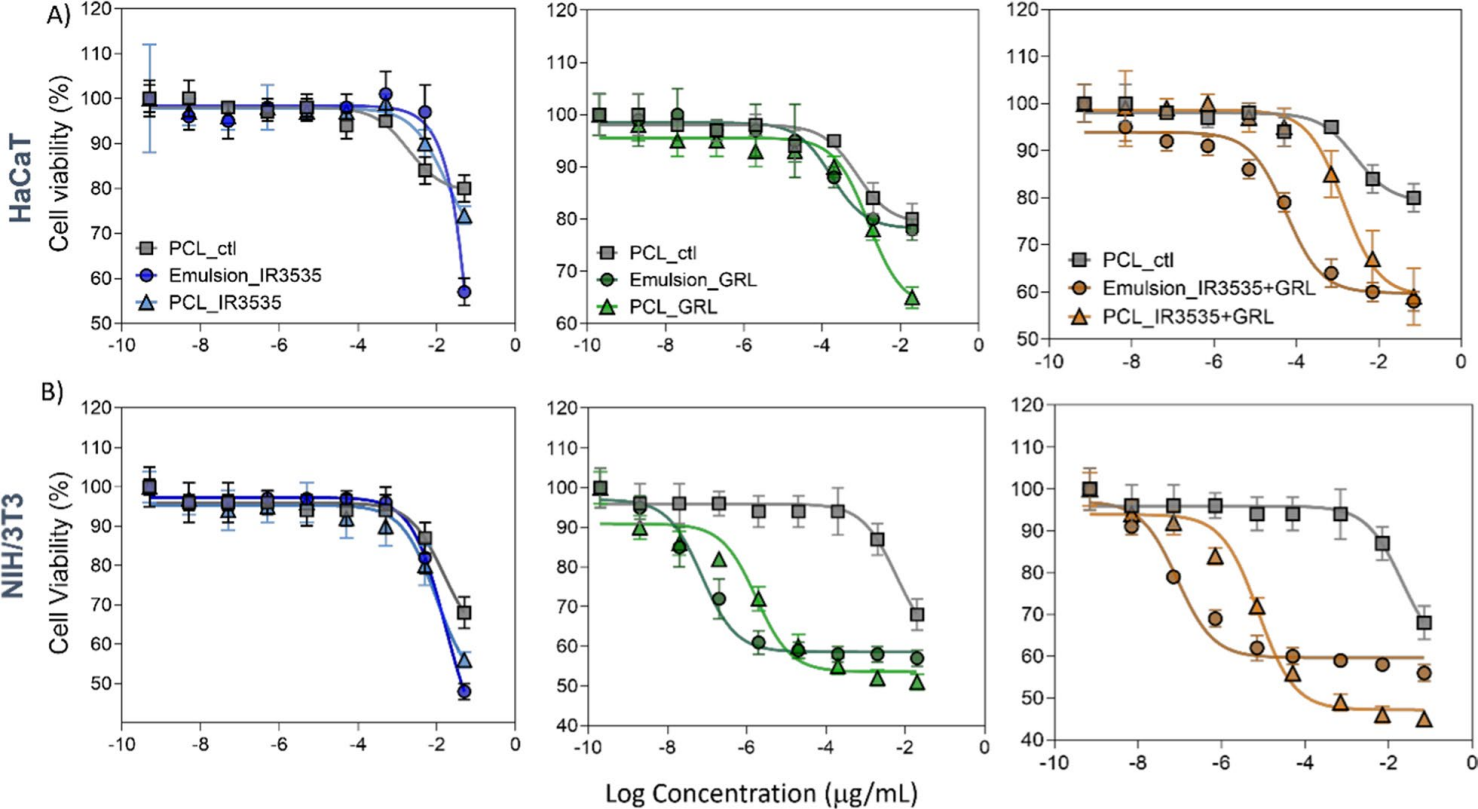
Cytotoxicity assay of non-encapsulated IR3535 (emulsion_IR3535), non-encapsulated GRL (emulsions_GRL), mixture of both compounds non-encapsulated (emulsion_IR3535 + GRL), PCL nanoparticles (PCL_ctl), PCL nanoparticles loading IR3535 (PCL_IR3535), PCL nanoparticles loading GRL (PCL_GRL), PCL nanoparticles loading both compouds (PCL_IR3535 + GRL) using **A** HaCaT cell line and **B** NIH-3T3 cell line after 24 h. Cell viability was evaluated by the MTT assay. The values represent the mean ± SD of three independent experiments

The formulation with IR3535 + GRL encapsulated in PCL nanoparticles at 500 mg/mL concentrations of IR3535 showed the most significant cytotoxic effects after 24 h with cell viability of 74 ± 2% and 46 ± 1% for HaCaT and 3T3 cells, respectively (Fig. 7A). GRL at 200 mg/mL showed cell viability of 65 ± 3% and 59 ± 6% for HaCaT and 3T3 cells, respectively (Fig. 7B). The reduced cell viability may be related to the nanoparticle composition and presence of the repellent active since reduced toxicity was observed for the control nanoparticles. Ridolfi et al. [66] studied the cytotoxic potential of solid lipid nanoparticles (SLNs) prepared with different lipids using the MTT method with the same cell lines, and verified the dose-dependent cytotoxicity of solid lipids used in nanoparticle synthesis. Mendes et al. [67] evaluated the MTT cytotoxicity of 3 nanostructured systems and observed that the number of nanoparticles in the dispersion negatively influenced cell viability, especially NLCs.

Previous studies also evaluated GRL cytotoxicity in skin tumor cells from Swiss albino mice, showing suppression of the Ras/Raf/ERK1/2 signaling pathway inducing apoptosis [68]. Another study evaluated GRL cytotoxicity in human tumor cell lines (HepG2 and A549) using MTT methods, demonstrating dose-dependent decreased viability [69]. Corroborating the results presented herein, Hazarika et al. [70] evaluated the cytotoxicity of a formulation containing clove oil, citronella, and lemongrass, showing decreased viability of human lung epithelial cells (L-132) with increasing concentrations of essential oils, especially between 500 and 1000 mg/mL [71] also reported dose-dependent GRL cytotoxicity in human lymphocyte cells, with 80% viable cells at a 2000 mg/mL concentration. Although the formulations showed more significant cytotoxicity towards the NIH-3T3 line, it should be noted that these repellent formulations focus on topical use. Therefore, the main objective is active volatilization, forming a vapor barrier on the skin due to a super concentrated local formulation. In addition, screening studies of polymeric and lipid nanoparticles on the skin found that PCL nanoparticles remained localized in lipid domains between the SC and corneocytes [70, 71].

**Fig. 8:**
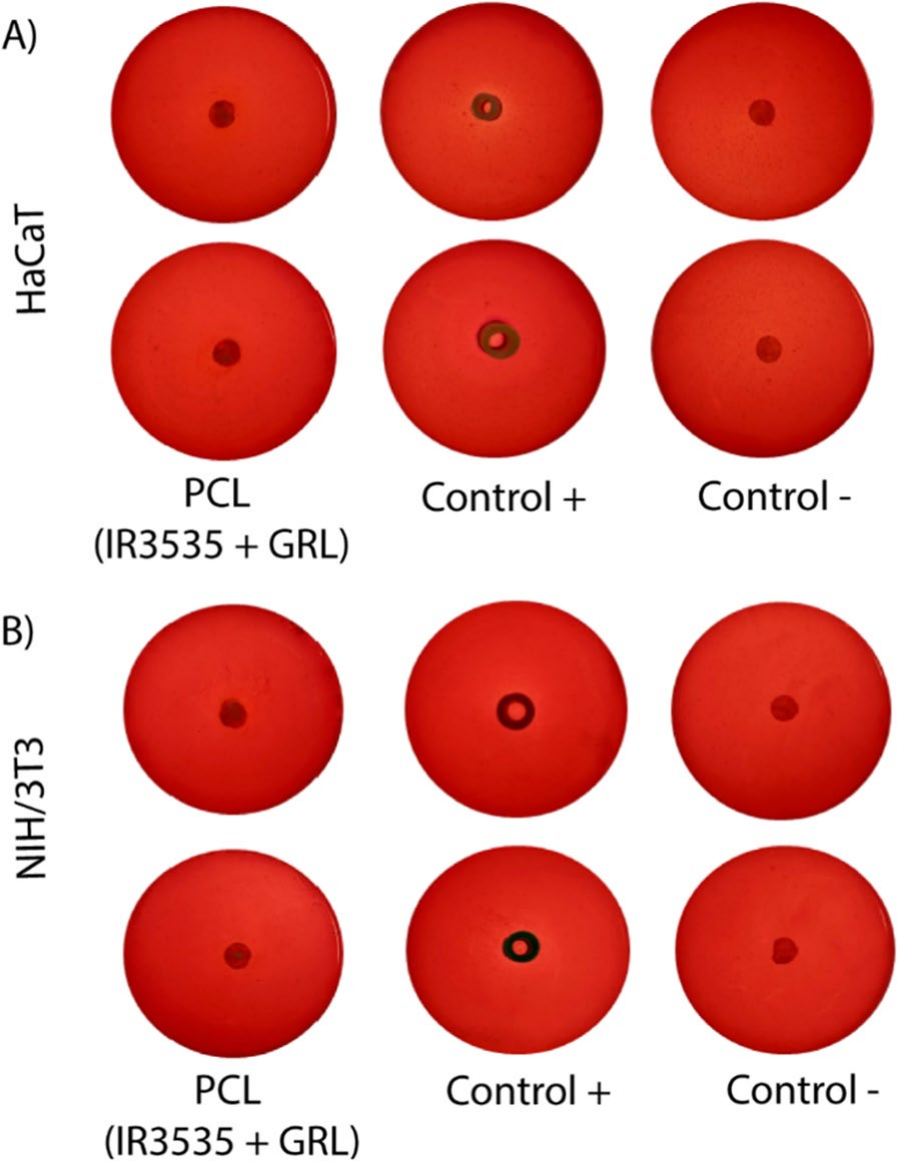
Cytotoxic assay of hydrogel containing PCL nanoparticles loading both compounds (PCL IR3535 + GRL) using **A** HaCaT cell line and **B** NIH-3T3 cell line after 24 h. Cell viability was evaluated by the halo formation. The values represent the mean ± SD of three independent experiments

The cytotoxicity results presented herein show that carrier systems based on PCL showed reduced toxic effects when compared to the emulsions, highlighting the potential of these systems as a tool to prolong protection times with lower actives concentrations. In addition, the gel formulations tested did not show significant cytotoxicity, without indication of a toxicity halo around or under the test substance, and with cells remaining intact without any morphological changes for both cell lines (Fig. 8). However, in this formulation the nanoparticles were incorporated or dispersed in a polymeric matrix (hydrogel), which likely reduced direct contact with the cells and, consequently, presented nontoxicity.

## Conclusions

This study developed a novel generation of repellent formulations containing PCL nanoparticle encapsulated IR3535 + GRL. Stability was achieved over 120 days with EE values of 60% and 99% for IR3535 and GRL, respectively, with a spherical morphology. Poloxamer hydrogels showed greater stability over the frequency range tested when compared to the other prepared systems. The release profiles of IR3535 were more evident in the PCL formulation incorporated in poloxamer 407 hydrogel compared to GRL. However, both actives exhibited low permeation after 24 h, indicating that the formulations (PCL nanoparticles and PCL in hydrogel) controlled the bioactive percutaneous absorption. Minor changes in the SC caused by interactions with formulations pose a small possible safety risk to the consumer. The cytotoxicity results show that the carrier systems based on PCL exhibited reduced toxicity when compared to emulsions, with potential for these systems to be used as a tool to prolong the protection time with reduced concentrations of repellent actives.

## Supplementary Information

**Additional file 1: Figure S1.** Images obtained from AFM, with a scanning window of 8 mm x 8 mm. A) average-sized frequency distribution histograms; B) Surface topography of PCLs (IR3535 5% + GRL 2%); C) 3D topography of the PCLs (IR3535 5% + GRL 2%); D) Surface topography of hydrogel containing PCLs (IR3535 5% + GRL 2%) and E) topography of hydrogel containing PCLs (IR3535 5% + GRL 2%). **Table S1. **Mathematical model parameters, such as release constant (k_2_); correlation coefficient (r^2^), and exponent of the release mechanism (n) after application of zero order, first order, Higuchi and Korsmeyer -Peppas models to the relase curve of IR3535 and GRL from the PCL nanoparticles formulation and hydrogel containing PCL nanoparticles formulation.

## Data Availability

The datasets used and/or analyzed during the current study are available from the corresponding author on reasonable request.
