# Supplementary material for: Repellent active ingredients encapsulated in polymeric nanoparticles: potential alternative formulations to control arboviruses
Source: J Nanobiotechnology. 2022 Dec 10;20:520. doi: 10.1186/s12951-022-01729-7 (PMC9741802; doi:10.1186/s12951-022-01729-7)
Supplement: Supplementary file 1 — Additional file 1: Figure S1. Images obtained from AFM, with a scanning window of 8 mm x 8 mm. A) average-sized frequency distribution histograms; B) Surface topography of PCLs (IR3535 5% + GRL 2%); C) 3D topography of the PCLs (IR3535 5% + GRL 2%); D) Surface topography of hydrogel containing PCLs (IR3535 5% + GRL 2%) and E) topography of hydrogel containing PCLs (IR3535 5% + GRL 2%). Table S1. Mathematical model parameters, such as release constant (k2); correlation coefficient (r2), and exponent of the release mechanism (n) after application of zero order, first order, Higuchi and Korsmeyer -Peppas models to the relase curve of IR3535 and GRL from the PCL nanoparticles formulation and hydrogel containing PCL nanoparticles formulation. [file 12951_2022_1729_MOESM1_ESM.docx]

**Additional information**

**REPELLENT ACTIVE INGREDIENTS ENCAPSULATED IN POLYMERIC NANOPARTICLES: POTENTIAL ALTERNATIVE FORMULATIONS TO CONTROL ARBOVIRUSES**

Daniele Carvalho Abrantes^1^, Carolina Barbara Rogerio^1^, Estefânia V. R Campos^1^, Tais Germano-Costa^2^, Aryane Alves Vigato^3^, Ian Pompermeyer Machado^3^, Anderson Ferreira Sepulveda^3^, Renata Lima^2^, Daniele Ribeiro de Araújo^3^, and Leonardo Fernandes Fraceto^1*^

^1^ São Paulo State University (UNESP), Institute of Science and Technology, Avenida Três de Março 511, Alto da Boa Vista, Sorocaba, São Paulo, 18087-180, Brazil

Jaboticabal, São Paulo, 14884-900, Brazil

^2^ Laboratory of Bioactivity Assessment and Toxicology of Nanomaterials, University of Sorocaba, Sorocaba, São Paulo, Brazil

^3^ Human and Natural Sciences Center, Federal University of ABC, Santo André, São Paulo, 09210-580, Brazil

**Figure**

**Figure S1.** Images obtained from AFM, with a scanning window of 8 μm x 8 μm. A) average-sized frequency distribution histograms; B) Surface topography of PCLs (IR3535 5% + GRL 2%); C) 3D topography of the PCLs (IR3535 5% + GRL 2%); D) Surface topography of hydrogel containing PCLs (IR3535 5% + GRL 2%) and E) topography of hydrogel containing PCLs (IR3535 5% + GRL 2%).

**Table**

**Table S1 –** Mathematical model parameters, such as release constant (k_2_); correlation coefficient (r^2^), and exponent of the release mechanism (n) after application of zero order, first order, Higuchi and Korsmeyer -Peppas models to the relase curve of IR3535 and GRL from the PCL nanoparticles formulation and hydrogel containing PCL nanoparticles formulation.

| **Release Kinetics** | Zero Order | | |  | First  Order | |  | Higuchi | |  | Korsmeyer-Peppas | | |
| --- | --- | --- | --- | --- | --- | --- | --- | --- | --- | --- | --- | --- | --- |
| **Formulations** | K_2_ r^2^ | | |  | K_2_ r^2^ | |  | K_2_ r^2^ | |  | K_2_ n | | r^2^ |
| **PCL_Suspension** | |  |  |  |  |  |  |  |  |  |  |  |  |
| IR3535 | 1.081 | | 0.9415 |  | 0.00251 | 0.7849 |  | 23.90 | **0.9896** |  | 0.6513 | 1.53 | 0.9889 |
| GRL | 0.0020 | | **0.9473** |  | 0.0005 | 0.7295 |  | 0.0476 | 0.9328 |  | 0.8467 | 1.18 | 0.8467 |
| **PCL_Gel** |  | |  |  |  |  |  |  |  |  |  |  |  |
| GRL | 0.0197 | | **0.9051** |  | 0.0050 | 0.6808 |  | 0.4549 | 0.8947 |  | 0.6858 | 1.45 | 0.8453 |
| IR3535 | 0.1590 | | 0.9346 |  | 0.00316 | 0.8520 |  | 3.697 | 0.9400 |  | 0.8737 | 1.14 | **0.9508** |
